# Polymeric Hydrogels for Controlled Release of Black Tea and Coffee Extracts for Topical Applications

**DOI:** 10.3390/gels7040174

**Published:** 2021-10-21

**Authors:** Pooja Makhija, Himanshu Kathuria, Gautam Sethi, Bert Grobben

**Affiliations:** 1 Department of Chemistry, National University of Singapore, 3 Science Drive 3, Singapore 117543, Singapore; 2 Department of Pharmacy, National University of Singapore, Singapore 117543, Singapore; himanshukathuria01@u.nus.edu; 3 Nusmetic Pvt Ltd., Makerspace, i4 Building, 3 Research Link, Singapore 117602, Singapore; 4 Department of Pharmacology, Yong Loo Lin School of Medicine, National University of Singapore, Blk MD3, 16 Medical Drive, Singapore 117600, Singapore; phcgs@nus.edu.sg; 5 Budding Innovations Pvt Ltd., 06-02 Jellicoe Rd, Singapore 208766, Singapore

**Keywords:** black tea, coffee, hydrogel, poly(acrylic acid), topical, tea milk interaction, coffee milk interaction

## Abstract

Tea and coffee are popular beverages. Both are also used in topical applications, such as ultraviolet (UV) protection, anti-aging, and wound healing. However, the impact of tea and coffee extract on skin cells is minimally explored. This study investigated the direct exposure of tea and coffee extract on skin cells using a 3-(4,5-dimethylthiazol-2-yl)-2,5-diphenyl-2H-tetrazolium bromide (MTT) assay. It was found that direct exposure of tea and coffee to skin cells can be toxic at a high dose on prolonged exposure (72 h). Therefore, it was hypothesized that a formulation providing a controlled release of tea and coffee could improve their skin compatibility. Thermally cross-linked poly(acrylic acid) hydrogels loaded with tea and coffee extracts (with and without milk) were formulated and optimized. The release profiles of these hydrogels were studied at varying loading efficiency. Milk addition with tea extract retarded the tea extract release from hydrogel while minimally affecting the coffee release. This effect was due to the molecular interaction of tea with milk components, showing changes in size, zeta potential, and polydispersity index. The release study best fitted the Korsmeyer–Peppas release model. Skin cells exposed to tea or coffee-loaded hydrogel showed normal skin cell morphology under fluorescence microscopic analysis. In conclusion, the hydrogels controlled the tea and coffee release and showed biocompatibility with skin cells. It can potentially be used for skin applications.

## 1. Introduction

Black tea (*Camellia sinensis var. assamica*) and coffee (*Coffea arabica*) are popular beverages, which provide several medicinal benefits. Black tea is industrially produced by the oxidation of green tea [1,2,3]. During the oxidation process, green tea catechins are partially converted into black tea polyphenols, such as theaflavins and thearubigins [1]. The dark orange color of black tea decoction is majorly due to theaflavins (theaflavin, theaflavin-3-O-gallate, theaflavin-3′-O-gallate, theaflavin-3,3′-O,O-digallate) [1]. It contains xanthines (caffeine, theobromine, and theophylline), catechins (epicatechin, epigallocatechin, epicatechin gallate, epigallocatechin gallate), and small amounts of isotheaflavin, neotheaflavin, theaflavate A, theaflavate B, isotheaflavin-30-O-gallate, neotheaflavate-3-O-gallate, theaflavic acids, theaflagallins, and methylated theaflavins [1,4,5,6,7]. Coffee is mainly obtained from *Coffea arabica*. Its main constituents are caffeic acid, chlorogenic acids, caffeine, cafestol, ferulic acid, pyrogallic acid, trigonelline, kahweol, hydroxycinnamic acid, 5-caffeoylquinic acid, nicotinic acid, pyrocatechol [8,9,10,11,12,13,14].

Several reports show that tea extract, coffee extracts, and their components have multiple bioactivities [10,15,16,17,18,19,20,21,22,23,24,25,26,27,28]. These can be used to treat various skin conditions like wounds, cellulitis, ultraviolet (UV) damage, acne, and aging [15,16,17,18,19,20,21]. The potential topical applications of these extracts are protection from oxidative stress and UV damage [22,23,24,25,26,27], anti-inflammatory [11,28,29], wound-healing [30,31,32,33], and sunscreen properties [34]. Green tea polyphenols were developed into microspheres by oxidative coupling, and loaded into polyvinyl alcohol/alginate hydrogel, later studied for diabetic wound healing [35,36]. A hair care formulation containing tea extract showed efficacy in UV damage prevention [37]. In a randomized, single-blind trial, a green tea extract with other extracts in hydrogel formulation showed antiacne and antiblotch efficacy, where hydrogel use over a period showed superior effects than 1% clindamycin gel [38]. ‘Silver needle white tea’ extract-treated wounds showed less inflammation and more angiogenesis than vehicle-treated wounds [31]. Green tea extracts showed wound healing potential in NIH3T3 fibroblast cells [39]. Coffee extracts are used in cosmetic products, which are commercially available [40,41]. Other studies show that an ointment containing coffee extract had wound-healing properties [42], and a gel containing green coffee and resveratrol protects dental caries [43]. Coffee extract incorporated in cellulose dressings showed enhanced antimicrobial activity [44]. Another group developed nanostructured lipid carriers encapsulating caffeine for the treatment of cellulitis [45,46].

The addition of milk to tea and coffee beverages has been an age-old practice that improves sensory acceptance. Several reports explored milk and its components, such as proteins, as a carrier for the delivery of tea extracts, coffee extracts, and its components. Tea polyphenols are known to interact with milk, and the potential role of milk as a carrier of tea components has been discussed in multiple studies [47,48,49,50,51,52,53,54]. Tea polyphenols have low chemical stability, and their interactions with milk proteins improve their stability [50,55,56]. In another study, liposomes prepared from milk phospholipids could protect ascorbic acid for seven weeks, a rapidly oxidizable molecule [57]. Milk-based liposomes have also been used to deliver epigallocatechin gallate (EGCG) [51]. Casein can bind EGCG without changing its activity [48,49] and selectivity [48], showing the appropriateness of casein micelles for polyphenol delivery [48,49]. In the case of coffee extracts, chlorogenic acid is the most studied interaction. It was found that chlorogenic acid binds with milk components, reducing antioxidant activity and bioavailability [58]. Chlorogenic acids interact with major milk proteins, i.e., α-casein, β-casein, κ-casein, α-lactalbumin, and β-lactoglobulin via different bonding mechanisms resulting in reduced antioxidant activity [59]. The other green coffee components, such as caffeic, ferulic, and chlorogenic acids, also interact with milk proteins [60].

Poly(acrylic acid) (PAA) polymer, known as carbomer, is a widely known safe ingredient used in pharmaceutical and cosmetic applications. Turkoglu et al. showed protection against UV damage using aqueous black tea extract loaded into a carbomer gel showed UV damage (erythema) protection in human subjects [61,62]. Recently, Ngamdokmai et al. showed carbopol 940 (a carbomer) gel with emulsion loaded with tea and coffee extracts for cellulite application [15]. Though commercially available carbomers can be formed into gels, they do not provide three-dimensional structure and rigidity for specialized applications such as facial film masks for topical use. Therefore, researchers explored a synthetic approach to prepare PAA hydrogels [63]. Zhu et al. prepared hybrid hydrogel with PAA and peptide for wound dressings with stimuli sensitivity and biodegradability [64].

Though the tea and coffee interaction with milk is known, the impact of milk on tea or coffee release from hydrogels or other delivery systems is not known. Therefore, in this study, the PAA hydrogel loaded with tea and coffee extracts was prepared, and later, the impact of milk on tea and coffee release was checked. First, the cell viability of tea and coffee extract on direct exposure to skin cells was measured. Then, PAA hydrogels loaded with tea and coffee extracts were prepared and optimized. Later, the loading efficiency and release kinetics of hydrogels were studied, along with the effect of milk addition. Finally, the skin cell morphology with exposure to tea and coffee-loaded hydrogels was evaluated.

## 2. Results and Discussion

### 2.1. Calibration Curves of Black Tea Extract and Coffee Extract

**Figure 1:**
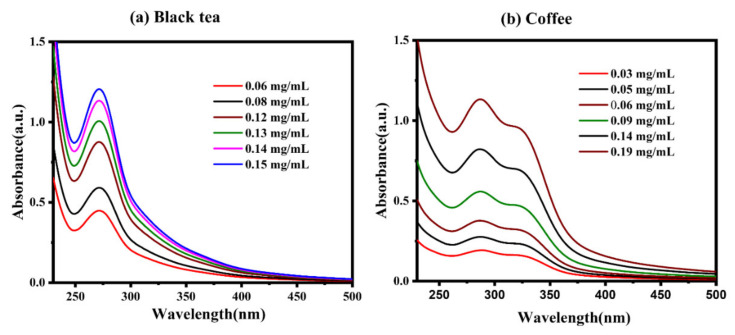
(**a**) UV-Vis spectra for calibration curve construction of (**a**) black tea extract in a range of 0.06–0.15 mg/mL. (**b**) coffee extract in a range of 0.03–0.19 mg/mL.

The yield of black tea dry extract was found in a range of 0.1% *w*/*w*. It is seen from the literature that the λ_max_ of known black tea extract and coffee components is in the range of 200–500 nm. Therefore, all the dilutions of the calibration curve of black tea extract (Figure 1a) and coffee extract (Figure 1b) are represented in the 200–500 nm range. These calibration curves were used to generate linear equations (Figure S1) to determine the values of unknown tea and coffee solution concentrations in release and entrapment studies.

### 2.2. Cell viability Assessment of Extracts

**Figure 2:**
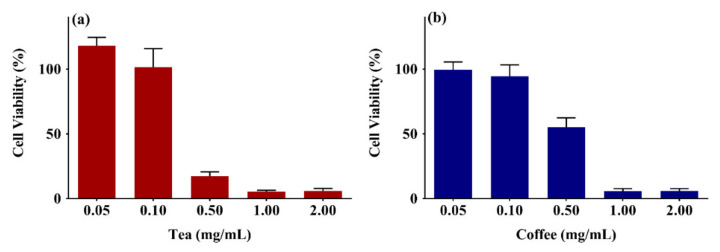
Cell viability study of (**a**) black tea extract, (**b**) coffee extracts on HaCaT cells using MTT assay. The results from three independent experiments (3 replicates in each experiment) are presented as mean ± standard deviation (SD).

There is a lack of understanding of the impact of black tea extract and coffee extracts on skin cells. Therefore, the cell viability of tea and coffee extracts was studied on the human skin keratinocytes (HaCaT cells) cells using 3-(4,5-dimethylthiazol-2-yl)-2,5-diphenyl-2H-tetrazolium bromide (MTT) assay as described previously [65]. The cell viability (%) was calculated using Equation (1). Figure 2a,b show the cell viability of HaCaT cells on being treated with 0.05–2 mg/mL of black tea and coffee extracts for 72 h, respectively. The cell viability of black tea extract was 118 ± 6%–6 ± 1.9%, whereas the cell viability for the coffee extract was 99.5 ± 6%–6 ± 1.8%. The cell viability values decreased drastically after the 0.1 mg/mL dose (tea extract) and 0.5 mg/mL dose (coffee extract). The tea and coffee extracts’ half-maximum inhibitory concentration (IC50) were 0.35 mg/mL and 0.56 mg/mL, respectively.

Previously the IC_50_ value of black tea extract has been reported as 0.56 mg/mL against the Caco-2 cells after 48 h, while the same extract was not cytotoxic to fibroblasts at this concentration [66]. Another study reported the IC_50_ values of black tea extracts as 1.4 × 10^−6^ mg/mL (HT-29 cells), 1.6 × 10^−6^ mg/mL (MCF-7 cells), >5 × 10^−3^ mg/mL (A549 cells), and >5 × 10^−3^ mg/mL (NIH-3T3 cells) [67]. A recent study reported the IC_50_ (48 h) of black tea extracts as 0.57 mg/mL and green tea extracts as 0.52 mg/mL against HepG2 cells [68], which are closer to IC_50_ obtained on HaCaT cells in our study.

Similarly, coffee extracts have also been evaluated for cytotoxicity against several in-vitro models. Coffea canephora var. robusta extracts also showed cytotoxicity against prostate cancer cells DU-145 [69]. Extracts from film removed from raw coffee beans were non-cytotoxic concentrations at and above 0.2 mg/mL against skin keratinocytes [70]. Methanolic extracts of low-grade green coffee beans and spent coffee beans were cytotoxic against leukemia cells (P388) [71]. Another study reported IC_50_ values (24 h) of roasted coffee extracts as 4.88 mg/mL against fibroblasts (L929) [72]. It is clear from the literature that the tea and coffee extracts are cytotoxic after a specific dose range, and the cytotoxicity also depends upon the source of the extract [73].

### 2.3. Preparation of Hydrogels

**Figure 3:**
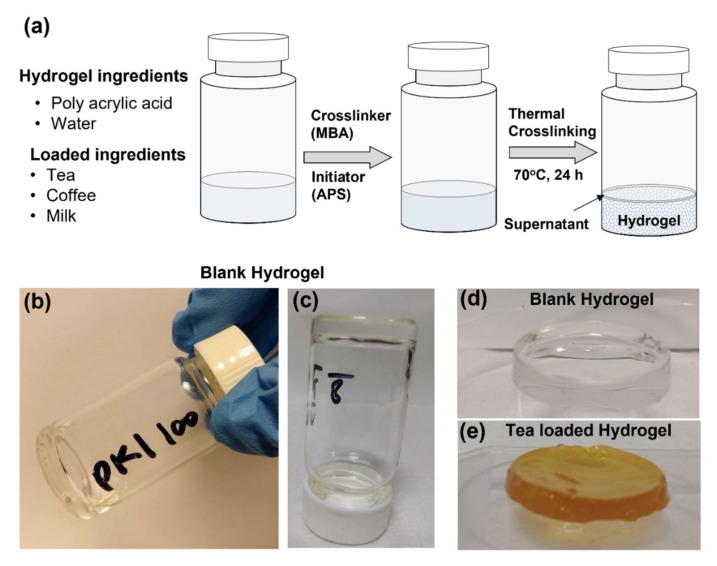
(**a**) Schematic of the hydrogel preparation process. (**b**–**d**) shows blank hydrogel formulae D (Table S1). (**e**) show the image of tea-loaded hydrogel.

The hydrogels were prepared from modified formulae from Joshi et al. [74]. The PAA amount was optimized to get the desired hydrogel properties (Figure S2, Table S1). Figure 3a shows the schematic of the hydrogel preparation process for the blank hydrogel, extract loaded, and extract with milk loaded hydrogels. The blank hydrogel was transparent, while tea and coffee hydrogels were brown in appearance. The milk addition reduced the intensity of the brown color and provided a milky appearance. The texture of hydrogels was rigid, adhesive, and firm.

### 2.4. Determination of Extract Loading in Hydrogels

**Figure 4:**
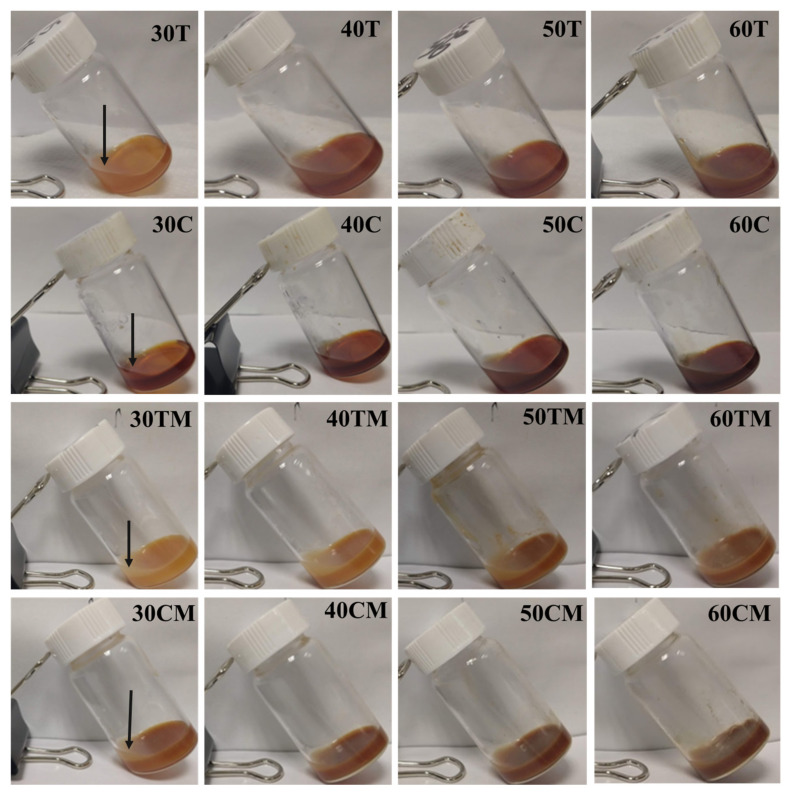
Physical observation of hydrogels after completion of the gelling period. Different hydrogels are shown here, incorporating tea or coffee with or without milk. The black arrow marks supernatant in the top layer of hydrogel.

Formulation D from Table S1 was selected for further incorporation of extracts during the gelling process. Incorporating a high amount (20 mg or more) of tea or coffee extracts interfered with the gelling process (Figure S3, Video S1). Therefore, different amounts of ammonium persulfate (APS) and N,N’-Methylenebisacrylamide (MBA) were optimized for complete gelling and achieving sufficient extract loading (Figure S3, Table S2). Finally, PAA, APS, and MBA quantities like formulation GT (Table S2) were selected to load different amounts of tea, tea-milk, coffee, and coffee-milk in hydrogels (Figure 4.) After 24 h of gelation, the supernatant liquid from individual gels (Figure 4, top layer of each hydrogel marked by black arrow) was collected. The supernatant was suitably diluted and analyzed using a Shimadzu UV-Vis spectrophotometer (UV-3600) in a wavelength range of 200–800 nm. The concentrations of the samples were determined using the calibration curve equation prepared using known quantities of the tea and coffee extract powder (Figure S1). The total amount of tea or coffee was expected to be the sum of the amount of tea or coffee remaining in the supernatant plus the amount entrapped in the hydrogel, considering no other factors contributed to the loss of tea or coffee. The extract loading in hydrogels of each hydrogel was estimated as loading efficiency, calculated using Equation (2). Table 1 shows the loading efficiencies of all 16 hydrogels prepared using varying amounts of dry extracts with and without milk. All values of the loading efficiencies were found to be in the range of 69–96%. Black tea extract-loaded hydrogels (30T, 40T, 50T, and 60T) showed efficiency from 90 ± 1%–92 ± 4%. On addition of milk, the values ranged from 75 ± 6%–84 ± 4% in formulation batches 30TM, 40TM, 50TM, and 60TM. The loading efficiencies of coffee extract hydrogels without milk were in the range of 88 ± 1%–89 ± 4%, whereas efficiencies of coffee extract hydrogels with milk were in the range of 84 ± 4%–82 ± 3%. However, milk addition did not impact the efficiency of the gelling system in a statistically significant manner. It is also clear that the loading efficiencies were not impacted by the original amount of tea or coffee extract added (3–60 mg) to the gelling system across similar formulations.

### 2.5. Release Profile and Area under the Curve (AUC) of Tea and Coffee Hydrogels with or without Milk

**Figure 5:**
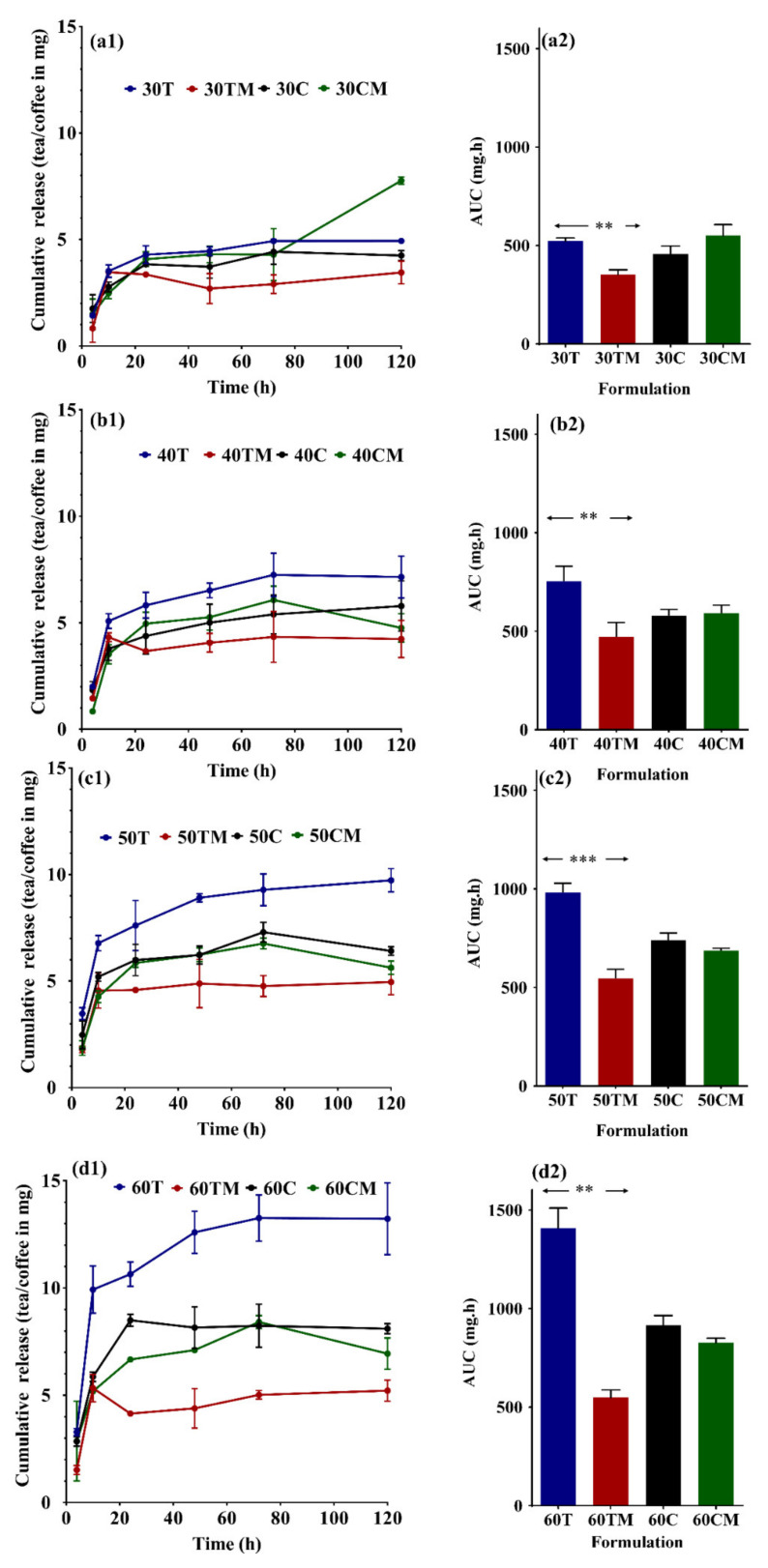
(**a1**–**d1**): Cumulative tea release over time from the different hydrogels for 120 h for different tea and coffee hydrogel formulations. (**a2**–**d2**): Area under the curve from the individual curve of cumulative tea release for 120 h the different hydrogels. The results from three independent experiments (3 replicates in each experiment) are presented as mean ± standard deviation (SD). Unpaired *t*-test with Welch’s correction was applied, ** indicate *p* < 0.01, *** indicate *p* < 0.001.

The diameter of each gel was 1.3 cm. Cumulative tea released after 4 h was 1.4 ± 0.1 mg for 30T, 2 ± 0.1 mg for 40T, 3.5 ± 0.3 mg for 50T, and 3.3 ± 1.7 mg for 60T. On the addition of milk, the cumulative tea release was 0.8 ± 0.7 mg (30TM), 1.5 ± 0.1 mg (40TM), 1.8 ± 0.2 mg (50TM), and 1.8 ± 0.2 mg (60TM). No definite trend can be deduced in the very initial hours of release. The cumulative tea release after 120 h (5 days) values were 4.9 ± 0.1 mg (30T), 7.2 ± 1.0 mg (40T), 9.7 ± 0.5 mg (50T), 13.2 ± 1.7 mg (60T), 3.4 ± 0.5 mg (30TM), 4.2 ± 0.9 mg (40TM), 5 ± 0.6 mg (50TM), and 5.2 ± 0.5 mg (60TM). The release profile of all eight formulations showed a similar trend, although the extent of release was different between the tea hydrogel versus tea-milk hydrogel. It appeared that milk hindered the release of tea from the hydrogels (Figure 5). The cumulative coffee release between the first (4 h) and last (120 h) studied release were 1.8 ± 0.7–4.2 ± 0.2 mg (30C), 1.8 ± 0.4–5.8 ± 1.2 mg (40C), 2.4 ± 0.7–6.4 ± 0.2 mg (50C), 2.9 ± 0.2–8.1 ± 0.2 mg (60C), 1.5 ± 0.7–7.8 ± 0.2 mg (30CM), 0.8 ± 0.4–4.8 ± 0.7 mg (40CM), 1.9 ± 0.3–5.6 ± 0.3 mg (50CM), and 2.9 ± 1.9–6.9 ± 0.7 mg (60CM). It appears that the release of coffee extract from the gel was not dependent on the presence or absence of milk (Figure 5). The release profile showed the best fitting into the Korsmeyer–Peppas model (Tables S3 and S4) in all except one formulation. This model works well with the formulations releasing the active through diffusion and erosion. The polymer surface looks eroded, and a decrease in color intensity of the brown surface (not shown). It is also noteworthy that the gels did not release the coffee or black tea extract entirely even after five days (physical observation by color).

The AUC of different formulations of tea, tea-milk, coffee, coffee-milk, and different formulation codes 30, 40, 50, and 60 were compared to highlight the impact of addition of milk. It appears that the release of coffee extract from the gel was not dependent on the presence or absence of milk. Figure 5 shows a significant difference between tea and tea-milk hydrogel AUC’s for all the different formula codes 30, 40, 50, and 60, whereas, in coffee versus coffee-milk hydrogels, none of the AUC’s were significant from each other. Few studies have reported that the tea catechins and milk proteins bind together, impacting the bioavailability [47,48,49,50,51,52,53,54,75]. Likewise, in this study, milk interaction with black tea has acted as a natural control release agent. However, for coffee, only interactions of chlorogenic acids with milk are known [58,59,60], which amounts to 4–11% *w*/*w* in instant coffee [76]. Our study also utilized freeze-dried (instant) coffee, so the levels of chlorogenic acid should be similar. Though the interactions of chlorogenic acid and milk are known, the milk addition did not further control the release of coffee. This can be attributed to stronger interaction of milk with tea components compared to milk interactions with coffee components.

Understanding the hydrogel’s release behavior is essential to estimate the amount of extract released in the system. There are different kinetic models based on mathematical calculations to calculate drug release from different formulations. In this study, release data from all 16 formulations were fitted into five types of release models: zero-order, first-order, Higuchi model, Hixson–Crowell, and Korsmeyer–Peppas model [77]. The R^2^ value closest to 1 was considered the measure of best fitting in the model. The mathematical analysis of the release data in Tables S3 and S4 showed the Korsmeyer–Peppas equation for all formulations except 30CM. Therefore, the Korsmeyer–Peppas model is more suitable for understanding the release of these hydrogel formulations than any other model. Furthermore, evidence in the literature points out the suitability of this model for formulations with polymer matrices such as hydrogel [77].

### 2.6. Interaction of Coffee and Tea with Milk

As observed in Section 2.5, the tea release from hydrogels was affected by the milk addition. Therefore, particle size and ζ-potentials were measured for black tea extract or coffee extract in water or milk-water (1:15) mixture. Table 2 shows the impact of milk on particle size and ζ-potentials. The addition of milk (−26.0 ± 0.404 mV) to tea (−10.5 ± 0.115 mV) brings the zeta potential of milk and tea solution to −20.0 ± 0.346 mV. However, the zeta potential of coffee (−10.8 ± 0.586 mV) and coffee with milk (−15.2 ± 0.351 mV) does not change much. The polydispersity index (PDI) of tea (0.318 ± 0.005) and coffee (0.384 ± 0.048) were higher than milk (0.248 ± 0.008), milk-tea (0.256 ± 0.008), and milk-coffee (0.280 ± 0.006) solutions. Thus, the PDI of tea or coffee with milk shows that milk reduces polydispersity, resulting from interaction with milk. The average hydrodynamic diameter (Zavg) of tea with milk was 295 ± 3 nm compared with 459 ± 13 nm for tea, indicating interactions leading to a smaller size. Similarly, Zavg of coffee with milk was 371 ± 6 nm compared with 467 ± 21 nm for coffee.

### 2.7. Morphological Analysis of Cells in the Presence of Extracts

**Figure 6:**
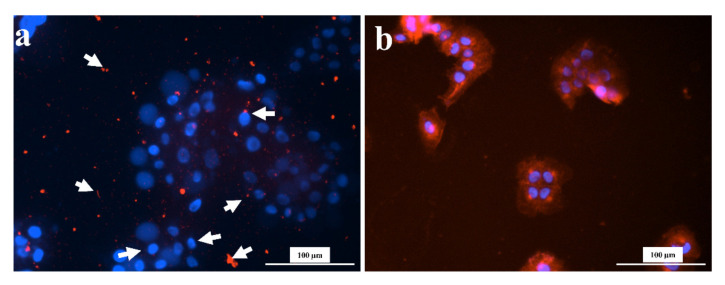
Morphological images (Cytation 3, 20X) of HaCaT cells after 12 h of treatment with 1.5 mg/mL of (**a**) tea and (**b**) coffee extracts. All images were recorded under the same conditions and presented with the same magnification for comparison.

In this section, the impact of tea and coffee on HaCaT cell viability was observed. This experiment was done to observe the impact of tea or coffee extracts on the HaCaT cell morphology. The extracts (tea or coffee) were added to HaCaT cells for 12 h. It was found that treating HaCaT cells with 1.5 mg/mL of tea or coffee extracts, even for a short treatment of 12 h, drastically impacted the morphology. Figure 6 shows that the coffee and black tea extract-treated cells are distorted in shape compared to control cells. In black tea extract-treated cells, even the cell membrane (red color) is not visible surrounding the nucleus. Instead, there are fragments of the membrane (indicated by arrows) shown in Figure 6. Previously, black tea theaflavins distorted gastric carcinoma cells (KATO-III) morphology [78]. Recently, black tea has been reported to distort the cell morphology of HepG2 cells (cell shrinking, membrane blebbing) [68]. However, such evidence is minimal for coffee extracts. One study reported co-administering coffee with anticancer agents but not coffee extract alone to distort MCF-7 cell morphology [79].

### 2.8. Morphological Analysis of Cells in the Presence of Hydrogels

**Figure 7:**
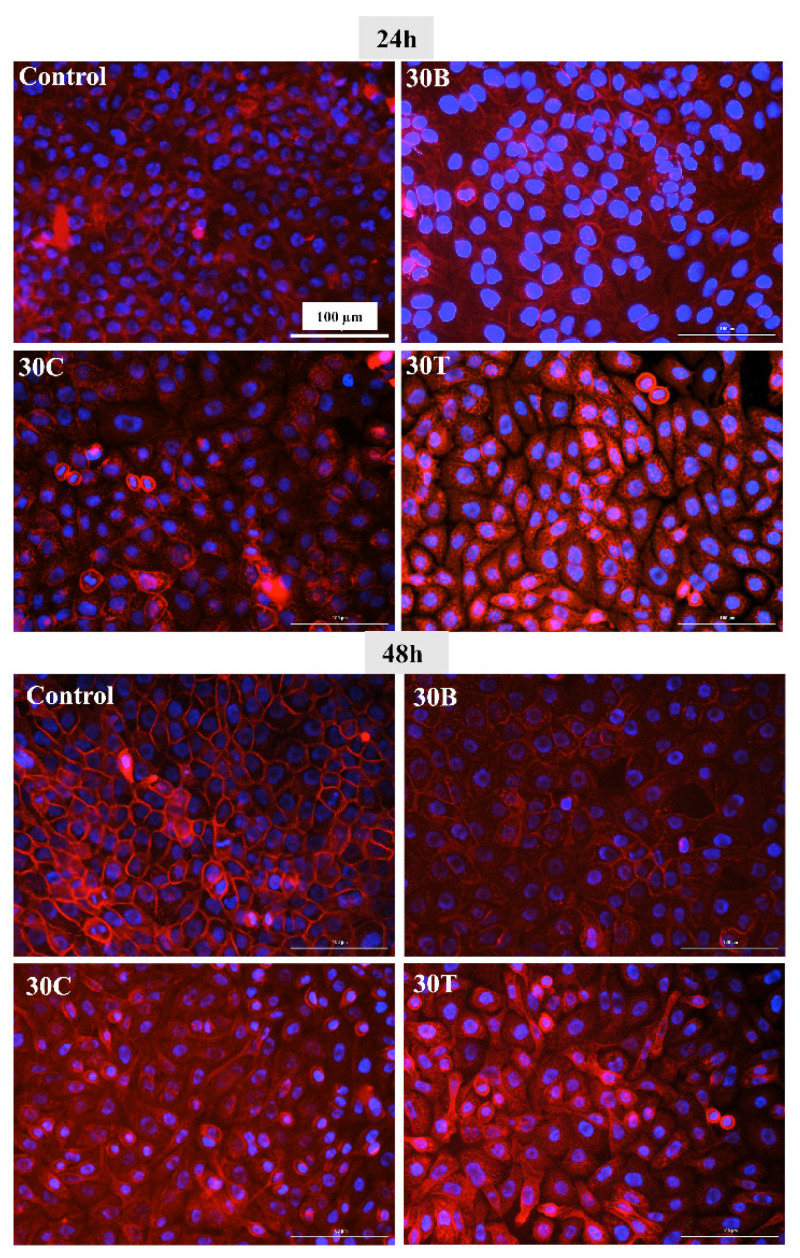
Morphological images (Cytation 3, 20X) of HaCaT cells after 24 h and 48 h of treatment with mini gel prepared from 100 µL of 30B, 30C, 30T, and control. All images were recorded under the same conditions and presented with the same magnification for comparison.

In this experiment, the impact of hydrogels on cell morphology was explored. Comparing control cells with cells in the vicinity of mini-gels (prepared from 100 µL of 30B, 30C, and 30T) shows that morphologically, there is not much difference across different treatments at both time points, 24 h and 48 h (Figure 7). The appearance of HaCaT cells in control, 30B, 30C, and 30T in the current study looks similar to that of the morphology reported previously [78,80,81].

### 2.9. Cell Viability Assessment of Media Incubated with Different Sizes of Hydrogel

**Figure 8:**
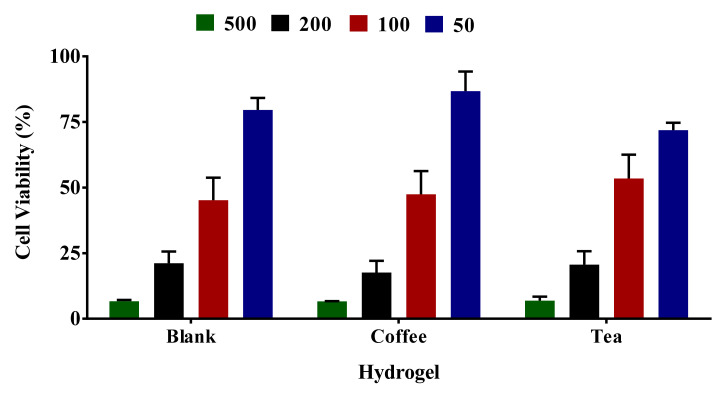
Different mini-gels of formulations were prepared for 24 h using different volumes (50–500 µL) of gelling solution. DMEM media was used to soak gels. HaCaT cells were treated for 24 h with DMEM media used for gel soaking. Impact of media leachate obtained after 48 h soaking of mini gels 30B, 30C, and 30T was evaluated by MTT assay. The results from three independent experiments (3 replicates in each experiment) are presented as mean ± standard deviation (SD).

In this study, media treated with different sizes of hydrogel (50–500 µL of 30T, 30C, and 30B) was used for cell viability testing. Figure 8 shows that the cell viability values were 79.6 ± 4.5%–6.6 ± 0.5% for 50–500 µL of blank hydrogel, 71.9 ± 2.8%–6.9 ± 1.5% for tea loaded hydrogel, and 86.7 ± 7.5%–6.6 ± 0.1% for coffee loaded hydrogel. The cell viability profile looks similar to all three formulations irrespective of the presence or absence of extracts. The cell viability values of mini-gels formed across different volumes of the three formulations (30T, 30C, 30B) were similar. These results indicate that the cell viability was not impacted by the presence of extracts in the formulation. However, this has also been evident from the data that blank gel shows the cell viability inhibition effects, which is evident from 79.6 ± 4.5% cell viability from a small volume of 50 µL of the blank hydrogel.

Our study did not determine the amount of unreacted APS in gel potentially leaching into the cellular system, given that the gels were washed with phosphate-buffered saline (PBS) and Dulbecco’s Modified Eagle Medium (DMEM) before setting up for leaching. It has been reported in the literature that exposing cells to APS concentrations of 100 mM or 500 mM lead for 2 h leads to a reduction in the fraction of live cells [82]. These findings have been confirmed in a different study [83]. One different aspect of MBA addition was highlighted in a study, where the time of complete utilization of MBA (in equivalent amount to the polymer) took three days [84] while the current study used only 24 h time. One study reported that MBA could cause toxicity with concentrations as low as 92 ± 31 µM in N1E-115 neuroblastoma [85]. In our study, 80.4 mM MBA was used in 2 mL gel preparation; however, the unreacted MBA was not qualified.

This study also observed the impact of media incubation on gel (Supplementary Materials, Figure S4). The hydrogel’s physical structure was distorted after incubation with media (Figure S4). The distorted structure shows the degradation of hydrogel in media. The color change to yellow (Figure S4b) indicates a decrease in pH, a standard media indicator of pH change. This physical change can be attributed to enzymatic degradation in media. Moreover, the color changes reveal the availability of more acidic groups upon enzymatic degradation. These suggest that PAA hydrogels are sensitive to biological media or enzymes, which can be studied further to prepare and optimize enzyme-sensitive hydrogels for biomedical applications. It also reveals that the reduced cell viability of HaCaT cells might be due to degradation products. However, the reduced cell viability is not a concern for topical application on intact skin as the hydrogels will not contact biological fluids, which can avoid degradation.

## 3. Conclusions

This study explored the direct exposure aqueous extract of commercially available tea and coffee in skin cells. The tea and coffee extracts were toxic on direct and prolonged exposure at high doses on skin cells. Polymeric hydrogel retarded the tea and coffee release. Adding milk into tea hydrogels further controlled the tea release. The gels were rigid, with adhesive properties and high loading efficiency of > 70% of tea or coffee extract. The tea and coffee hydrogels were biocompatible with skin cells without any morphological changes. Overall, the tea and coffee-loaded hydrogel showed potential in the controlled delivery of tea and coffee extracts for skin application. The hydrogel properties were suitable to make bioadhesive mask for skin applications.

## 4. Materials and Methods

### 4.1. Materials

Black tea leaves were purchased from the Red label brand. Coffee was obtained as commercial use granulated lyophilized powder was obtained from Boncafe brand. UHT milk was obtained from the NTUC FairPrice brand. PAA 450 kDa, MBA, APS, and MTT were purchased from Sigma Aldrich, St. Louis, MO, USA. HaCaT cells were bought from the American Type Culture Collection (ATCC), Manassas, VA, USA. DMEM and fetal bovine serum were bought from Biowest, France. Penicillin-Streptomycin (100×) was purchased from Thermo Fisher Scientific, Waltham, MA, ‎USA.

### 4.2. Black Tea Dry Extract Preparation

Black tea extract preparation was done using a slight modification of the previously described method [86]. First, 8 g of black tea leaves were soaked in 400 mL deionized (DI) water maintained at a temperature of 28–30 °C for 30 min. Then, the extract obtained was filtered, frozen at −80 °C, and freeze-dried to get a dry extract. Finally, the dried black tea extract was weighed and stored at −20 °C for further use.

### 4.3. Cell Viability Assessment of Tea and Coffee Extracts

The HaCaT cell line was subcultured in DMEM medium supplemented with 20% fetal bovine serum, 1% penicillin-streptomycin, and incubated in the presence of 5% CO_2_ and 95% humidity at 37 °C temperature. A cell suspension of 5 × 10^3^ cells in 100 µL of DMEM medium was seeded into each well of the 96-well plate and incubated overnight inside an incubator. These wells were treated with 100 µL of DMEM medium containing tea and coffee extracts in such a way that the concentration of tea or coffee extracts was 0.025 mg/mL, 0.05 mg/mL, 0.075 mg/mL, 0.1 mg/mL, 0.5 mg/mL, 1 mg/mL, and 2 mg/mL inside different wells. The cells inside the control well were not exposed to any extract but only had the DMEM medium. After adding extracts, plates were incubated for 72 h, followed by the addition of MTT reagent solution (20 µL, 5 mg/mL, Sigma Aldrich, USA) to each well and incubated again for 3 h. The excess medium was aspirated, and 100 µL dimethyl sulfoxide (DMSO) was added to each well. The optical density measurements (OD) were performed using a microplate reader (SPARK™ 10M, Tecan, Männedorf, Switzerland) at 570 nm. Relative cell viability was derived from the following Equation (1).

(1) $$\mathrm{Cell}\;\mathrm{viability}\;\left(\%\right)=\left(\frac{\mathrm{OD}\;\mathrm{of}\;\mathrm{treated}\;\mathrm{cells}}{\mathrm{OD}\;\mathrm{of}\;\mathrm{media}\;\mathrm{only}\;\mathrm{controls}}\right)\times 100$$

### 4.4. Preparation of Hydrogels

The PAA hydrogel formulation was adapted from Joshi et al. [74]. For the preparation of hydrogels without milk, 150 mg of PAA was first dissolved in 2 mL of DI water or extract solution in DI water for 3–4 h. After the complete dissolution of PAA, 24.8 mg of MBA and 50 mg of APS were added. The mixture was stirred until dissolved. Next, the mixture was transferred to a 20 mL glass vial, capped, and kept at 70 °C for 24 h for gelation (Table 3). For preparing tea or coffee, hydrogels with milk, the volumes of water, and milk were taken, as per Table 3. Black tea dry extract or coffee extract (Table 3) was added to the water–milk solution. This mixture was slowly pipetted to ensure uniform mixing of tea or coffee components with the water-milk solution while avoiding bubble formation. Then, 150 mg of PAA was dissolved in 2 mL of water–milk–tea or water–milk–coffee solution. As described above, MBA and APS addition were followed by gelation similar to the steps for preparing hydrogels without milk.

### 4.5. Loading Efficiency of Tea and Coffee in Hydrogels

After 24 h of gelation, the supernatant liquid from individual gels prepared (using formulation from Table 3) was collected in separate vials. Each sample was suitably diluted and analyzed using a Shimadzu UV-Vis spectrophotometer (UV-3600) in a 200–800 nm wavelength range. The concentrations of the samples were determined using the calibration curve prepared using known quantities of the black tea and coffee extract (Figure S2). The calculated loading of tea or coffee extract in the hydrogel was calculated by subtracting the amount of tea or coffee left in the supernatant on the top of the hydrogel from the total amount of tea or coffee extract loaded hydrogel solution before keeping for gelation. Loading efficiency (%) is calculated by Equation (2) below.

(2) $$\mathrm{Loading}\;\mathrm{efficiency}\;\left(\%\right)=\frac{\left(\mathrm{Original}\;\mathrm{amount}\;\mathrm{added}-\mathrm{Amount}\;\mathrm{in}\;\mathrm{supernatant}\right)}{\left(\mathrm{Original}\;\mathrm{amount}\;\mathrm{added}\;\right)}\times 100\;$$

### 4.6. Release Study of the Hydrogels

After collecting the supernatant, the surface of the gel was washed three times with DI water and kept upside down overnight to drain any traces of left-over water. For the release study, 10 mL of water was added to the gel’s top while still attached to the 20 mL glass vial bottom. At different time points (4 h, 8 h, 12 h, 24 h, 48 h, 72 h, 96 h, 120 h), 1–2 mL of sample was withdrawn and replaced with an equal volume of fresh DI water. Each sample was suitably diluted and analyzed using a Shimadzu UV/Vis spectrophotometer (UV-3600) in a 200–800 nm wavelength range. The concentrations of the samples were determined using the calibration curve prepared using known quantities of the tea and coffee extract powder.

### 4.7. Interaction of Tea or Coffee with Milk

The size and zeta potential (ζ-potential) for tea extract or coffee extract with or without milk were obtained to understand interactions. Hence, 30 mg of tea or coffee powder was dissolved in 2 mL of water or 2 mL of milk-water (1:15) mixture as prepared for 30TM/30T or 30CM/30C (Table 3). The solutions were diluted as required to carry out particle size and ζ-potentials measurements using Malvern Zetasizer Nano-ZS90.

### 4.8. Morphological Analysis of Cells

Briefly, 100 µL of 30T, 30C, and 30B hydrogel solution before gelling were poured in the corner of the 6-well plate and allowed to gel for 24 h. After 24 h, the gel surface was sterilized under UV for 15 min, washed with 1mL PBS, quickly followed by 1mL of DMEM. Then, 5 × 10^5^ HaCaT cells were seeded in the individual wells (sparing the part containing the gel). Later, 3 mL of media was added to each well. The plate was incubated for 24 h and 48 h in an incubator. After 24 h and 48 h, the media was removed from each well. Then, 1 mL of media containing CellMask™, orange plasma membrane stain (Thermofisher Scientific), and NucBlue™ live readyprobes™ reagent (Thermofisher Scientific) was added to the cells and kept under dark for 25–30 min. After incubation, the media with stains was removed, and the stained cells were washed gently with PBS 3 times. Later, 2 mL of PBS was added to cover the cells and prevent drying until imaged. Cells were photographed with a 20× objective lens on a Cytation 3 Cell Imaging Multi-Mode Reader (BioTek Instruments, Inc., Winooski, VT, USA) using a blue filter (377,447) and a red filter (586,647).

For the morphological analysis of cells with tea and coffee extracts, the cells were treated with 1.5 mg/mL of tea or coffee extract prepared in DMEM for 12 h. After completion of treatment time, staining and imaging steps were the same as above.

### 4.9. Cell Viability Assessment of Media Incubated with Different Sizes of Hydrogel

The media treated with different sizes of hydrogel (after 48 h incubation in media) was used for cell viability testing. The hydrogels were prepared using gelling solutions of 30T, 30C, and 30B (Table 3). Then, varying sizes of hydrogel were prepared by different volumes (50 µL, 100 µL, 200 µL, 500 µL) of 30T, 30C, and 30B (Table 3). The gelling solutions were poured into a 24-well plate and allowed to gel at 70 °C for 24 h. After 24 h, mini-gels (50 µL, 100 µL, 200 µL, 500 µL volume) were formed, and the gel surface was sterilized under UV light for 15 min. Then the surface was quickly washed with 1 mL PBS, followed by 1 mL of DMEM. This procedure was repeated three times to ensure no unreacted components from the surface leach out in the medium during the incubation process. After this, the gels were kept in contact with 2 mL of DMEM medium for 48 h. After 48 h, the medium was removed and stored at −20 °C until studied. The HaCaT cells were subcultured and seeded into each well of the 96-well plate as described in the previous section. After attachment, the cells were treated with leachate. The cells inside the control well were exposed to fresh media. After adding extracts, plates were incubated for 24 h, followed by the addition of MTT reagent solution (20 µL, 5 mg/mL, Sigma Aldrich, USA) to each well and incubated again for 3 h. Finally, the excess medium was aspirated, and DMSO (100 µL) was added to each well. The optical density measurements (OD) were performed using a microplate reader (SPARK™ 10M, Tecan, Switzerland) at 570 nm. Relative cell viability was derived from Equation (1).

The cell viability assessment of hydrogel could not be performed using the general MTT assay protocol. The MTT assay is based on the optical density determination of formazan crystals. The reduction in cell viability is understood by the reduction in the optical density of formazan crystals. However, in the case of hydrogels, the hydrogel surface adsorbs formazan crystals during MTT incubation, which could not be dissolved even on the addition of DMSO, causing a false reduction in cell viability. Therefore, considering the limitation of the assay, an MTT assay was performed using media incubated with hydrogels.

### 4.10. Statistical Analysis

An unpaired t-test with Welch’s correction was used for analyzing the data. All data are presented as a mean of 1–3 determinations ± standard deviation of the mean. * (*p* < 0.05), ** (*p* < 0.01), *** (*p* < 0.001) or **** (*p* < 0.0001) show statistically significant differences compared to control unless otherwise stated.

## Figures and Tables

**Table 1:** Loading efficiencies of different hydrogel formulations.

Code	Loading Efficiency (%)	Code	Loading Efficiency (%)
30T	90 ± 1	30TM	75 ± 6
40T	90 ± 2	40TM	78 ± 8
50T	91 ± 2	50TM	80 ± 7
60T	92 ± 4	60TM	84 ± 4
30C	88 ± 1	30CM	84 ± 4
40C	87 ± 2	40CM	78 ± 6
50C	86 ± 2	50CM	83 ± 3
60C	89 ± 4	60CM	82 ± 3

T = Black tea extract; TM = Black tea extract and milk, C = coffee extract, CM = coffee extract and milk, B = blank formulation without milk, BM = blank formulation with milk; 30 = 30 mg of extract; 40 = 40 mg of extract; 50 = 50 mg of extract; 60 = 60 mg of extract. The results from three independent experiments (3 replicates in each experiment) are presented as mean ± standard deviation (S.D.). Unpaired t-test with Welch’s correction was applied.

**Table 2:** Effect of milk addition on the zeta potential and Zavg (d.nm) of tea and coffee extracts.

Sample	Zeta Potential (mV)	Zavg (d.nm)	PDI
Milk	−26.0 ± 0.404	350 ± 3	0.248 ± 0.008
Tea	−10.5 ± 0.115	459 ± 13	0.318 ± 0.005
Coffee	−10.8 ± 0.586	467 ± 21	0.384 ± 0.048
Tea + Milk	−20.0 ± 0.346	295 ± 3	0.256 ± 0.008
Coffee + Milk	−15.2 ± 0.351	371 ± 6	0.280 ± 0.006

Zavg = average hydrodynamic diameter; PDI = Polydispersity Index.

**Table 3:** Formulation of PAA hydrogels loaded with black tea dry extract and coffee extract.

Formulation Code	Tea	Coffee	Water	Milk
B	-	-	2 mL	-
30T	30 mg	-	2 mL	-
40T	40 mg	-	2 mL	-
50T	50 mg	-	2 mL	-
30T	60 mg	-	2 mL	-
30C	-	30 mg	2 mL	-
40C	-	40 mg	2 mL	-
50C	-	50 mg	2 mL	-
60C	-	60 mg	2 mL	-
BM	-	-	1.75 mL	0.25 mL
30TM	30 mg	-	1.875 mL	0.125 mL
40TM	40 mg	-	1.833 mL	0.167 mL
50TM	50 mg	-	1.792 mL	0.208 mL
60TM	60 mg	-	1.75 mL	0.25 mL
30CM	-	30 mg	1.875 mL	0.125 mL
40CM	-	40 mg	1.833 mL	0.167 mL
50CM	-	50 mg	1.792 mL	0.208 mL
60CM	-	60 mg	1.75 mL	0.25 mL

T = Black tea extract; TM = Black tea extract and milk, C = coffee extract, CM = coffee extract and milk, B = blank formulation without milk, BM = blank formulation with milk; 30 = 30 mg of extract; 40 = 40 mg of extract; 50 = 50 mg of extract; 60 = 60 mg of extract.

## Data Availability

The data presented in this study are available on request from the corresponding author.

## Supplementary Materials

The following are available online at https://www.mdpi.com/article/10.3390/gels7040174/s1, Figure S1: Calibration curve of black tea and coffee extract, Figure S2: Physical appearances of blank hydrogels, Figure S3: Physical observation of gelling during optimization Figure S4: Effect of media on hydrogel integrity, Table S1: Optimization of PAA amount based on physical properties, Table S2: Optimization of Black tea extract loading with APS and MBA amounts, Table S3: Model-fitting of the release of tea and tea-milk hydrogels, Table S4: Model-fitting of the release of coffee and coffee-milk hydrogels, Video S1: Incomplete Hydrogel due to black tea addition.
