# Supplementary material for: Polymeric Hydrogels for Controlled Release of Black Tea and Coffee Extracts for Topical Applications
Source: Gels. 2021 Oct 21;7(4):174. doi: 10.3390/gels7040174 (PMC8544385; doi:10.3390/gels7040174)
Supplement: Supplementary file 1 [file gels-07-00174-s001.zip › 210922 Supplemantary information-R1.pdf]

gels

Polymeric Hydrogels for Controlled Release of Tea and Coffee extracts for Topical Applications

Pooja Makhija[1*], Himanshu Kathuria[2,3], Gautam Sethi[4], Bert Grobben[5**]

[1*] Department of Chemistry, National University of Singapore, 3 Science Drive 3, 117543, Singapore. pooja@u.nus.edu
[2] Department of Pharmacy, National University of Singapore, Singapore 117543, Republic of Singapore. himanshukathuria01@u.nus.edu
[3] Nusmetic Pvt Ltd, Makerspace, i4 building, 3 Research Link Singapore 117602, Republic of Singapore
[4] Department of Pharmacology, Yong Loo Lin School of Medicine, National University of Singapore, Blk MD3, 16 Medical Drive, 117600, Singapore
[5**] Budding Innovations Pvt Ltd, 06-02 Jellicoe Rd, Singapore 208766. bert.grobben@budinno.com

Correspondence: Bert Grobeen[**] and Pooja Makhija[*]

S1. Calibration graph of tea and coffee extract

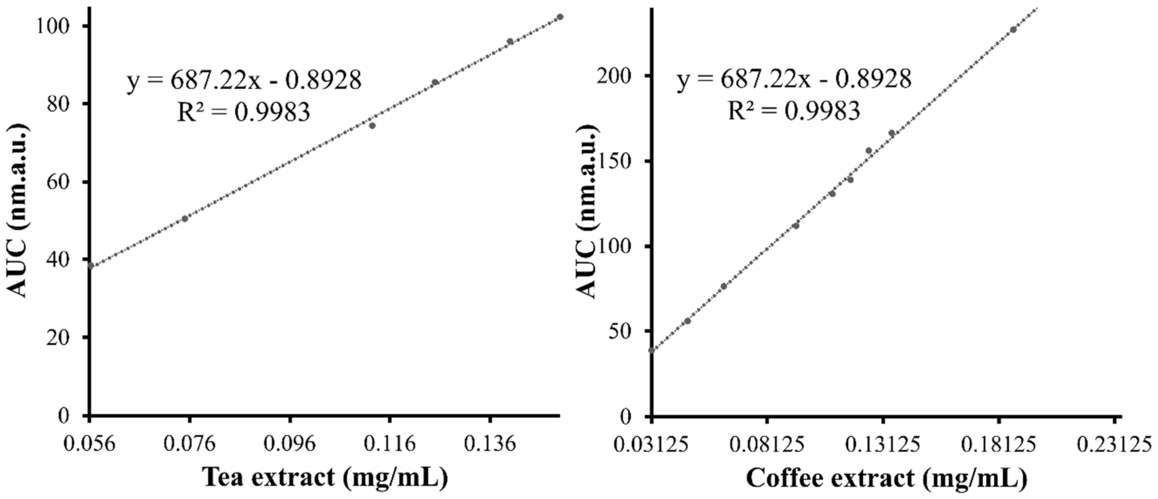

Figure S1. Calibration curve of black tea extract (left) in a range of 0.056 mg/L – 0.15 mg/mL, and calibration curve of coffee extract (right) in a range of 0.031 mg/L – 0.188 mg/mL.

S2. Optimization of the blank hydrogel formulation

First, poly(acrylic acid) 450 kDa (PAA) was dissolved in 2 mL of DI water for 3-4 h. After the complete dissolution, methylenebisacrylamide (MBA) was added to the solution and stirred until complete dissolution. Then, ammonium persulfate (APS) was then added and stirred until dissolved. Next, the mixture was transferred to a

mold if required and kept at 68-70 °C for 24 h for gelation. Table S1 shows the various amounts of PAA tested to get the desired blank formulation. Figure S2 shows the physical appearance of gels formed based on formulas from Table S1. Gel D was selected for final optimization with tea and coffee extracts.

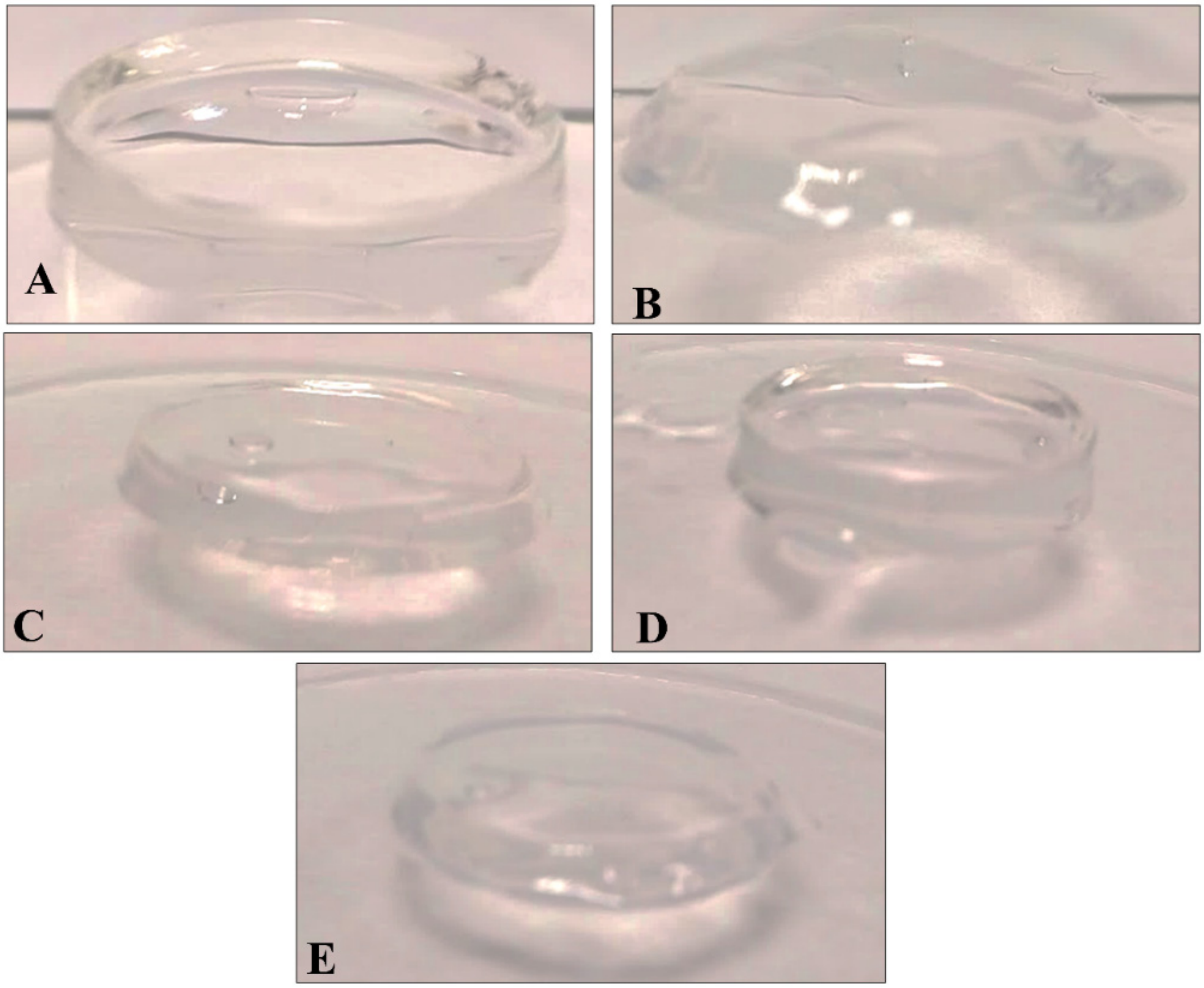

Figure S2. Physical appearances of blank hydrogels formed using different amounts of PAA as per table S1.

Table S1. Optimization of PAA amount based on physical properties. The amounts of MBA (6.2 mg), APS (12.5 mg) and water (2 mL) were kept contstant.

Code	Polymer	Characteristics
A	50 mg	Holds shape, firm
B	75 mg	Breaks on holding
C	100 mg	Does not hold shape, does not break on holding
D	150 mg	Better holding than A, firm
E	200 mg	Very sticky, firm

S3. Optimization of tea extract loading with APS and MBA amounts

The formulation D (Figure S2, Table S1) was selected based on its physical features. In AT, it was feasible to load 4 mg of tea with complete gelation. The complete gelling did not happen on increasing the tea amount from 20 mg (Video S1). Therefore, the higher amount of APS and MBA to load the tea amounts 20 mg or more was studied. Table S2 and Figure S3 show the various amounts of APS, MBA varied to increase extract loading in the formulation. Complete gelling was characterized by visual observation of gel thickness and only tea extract as supernatant on the top of the gel. The gelling was considered incomplete by observing the presence of polymer in the supernatant (more viscous, low flow) and uneven gel surface. FT and GT show clearer supernatant as compared to ET. However, the amount of APS and MBA from GT was chosen to load a higher tea and coffee extract concentration.

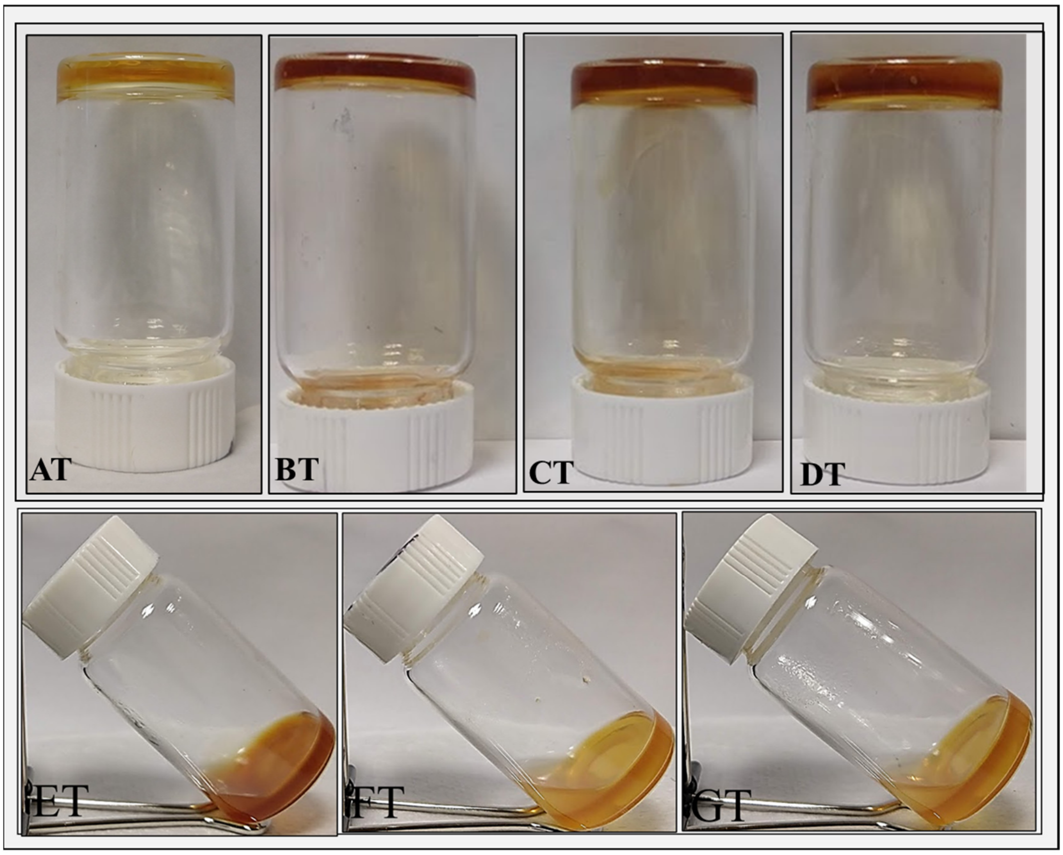

Figure S3. Physical observation of gelling outcomes during optimization of tea extract loading with changing APS and MBA amounts

Table S2. Optimization of tea extract loading with APS and MBA amounts with 150 mg PAA.

Code	MBA (mg)	APS (mg)	D factor#	Tea (2 mL)	Characteristics
AT	6.2	12.5	1X	2 mg/mL	Gelling complete
BT	6.2	12.5	1X	10 mg/mL	Incomplete gelling
CT	12.4	25	2X	10 mg/mL	Incomplete gelling
DT	18.6	37.5	3X	8 mg/mL	Incomplete gelling
ET	18.	37.5	3X	10 mg/mL	Incomplete gelling
FT	21.7	43.8	3.5X	10 mg/mL	Complete gelling
GT	24.8	50	4X	10 mg/mL	Complete gelling

AT-GT are the codes for various batches formed during the extract loading optimization. # D factor shows the times higher amount of crosslinker and initiator used based on formulae D of table S1.

S4. Model-fitting of tea or coffee extract release data

Understanding the hydrogel's release behavior is essential to estimate the amount of extract released in the system. There are different kinetic models based on mathematical calculations to calculate drug release from different formulations. In this study, release data from all 16 formulations were fitted into five types of release models [1] (Equation S1, Equation S2, Equation S3, Equation S4, and Equation S5). The R^2 value closest to 1 was considered the measure of best fitting in the model. Equation S1 represents the zero-order model where Ct is the concentration of drug released during the time t, C0 is the concentration of active released at t = 0 (it is considered zero), and K is the zero-order constant1.

$$\mathrm{Ct} = \mathrm{C0} + \mathrm{Kt} \qquad \text{(Equation S1)}$$

Equation S2 represents the first-order model where Qt is the amount of active released during the time t, Q0 is the concentration of active released at t = 0, and K is the first-order constant[1].

$$\log Q_t = \log Q_0 + \frac{Kt}{2.303} \qquad \text{(Equation S2)}$$

Equation S3 represents the Korsmeyer-Peppas model where M_∞ is the amount of drug at equilibrium; Mi is the amount of active released during the time t, K is the constant, and n is the release exponent[1].

$$\frac{M_i}{M_\infty} = Kt^n \qquad \text{(Equation S3)}$$

Equation S4 represents Higuchi release model; Q is the amount of active released at time t, K_H is the rate constant[1].

$$Q = K_H\sqrt{t} \qquad \text{(Equation S4)}$$

Equation S5 represents the Hixon-Crowell release model where W_0 is the initial amount of active, W_i is the remaining amount of active at time t, and K_{HC} is the constant[1].

$$\sqrt[3]{W_0} = \sqrt[3]{W_i} + K_{HC}t \qquad \text{(Equation S5)}$$

Table S3. Regression coefficient (R^2) obtained from the different model-fitting of the release profile of tea and tea-milk hydrogels

Code	30T	30TM	40T	40TM	50T	50TM	60T	60TM
Zero-order	0.5	0.2	0.5	0.3	0.6	0.5	0.5	0.6
1st order	0.5	0.5	0.5	0.5	0.5	0.5	0.5	0.5
Korsmeyer Peppas	0.9	0.6	0.9	0.7	1.0	0.8	0.9	1.0
Higuchi	0.8	0.5	0.8	0.5	0.9	0.7	0.7	0.9
Hixson-Crowell	0.001	0.001	0.001	0.001	0.001	0.001	0.001	0.001

T = tea extract; TM = tea extract and milk ; 30 = 30 mg of extract; 40 = 40 mg of extract; 50 = 50 mg of extract; 60 = 60 mg of extract

Table S4. Regression coefficient (R^2) obtained from the different model-fitting of the release profile of coffee and coffee-milk hydrogels

Code	30C	30CM	40C	40CM	50C	50CM	60C	60CM
Zero-order	0.5	0.6	0.3	0.6	0.5	0.4	0.4	0.4
1st order	0.5	0.5	0.5	0.5	0.5	0.5	0.5	0.5
Korsmeyer Peppas	0.9	0.8	0.8	1.0	0.9	0.9	0.9	0.9
Higuchi	0.7	0.9	0.6	0.8	0.7	0.7	0.7	0.7
Hixson-Crowell	0.001	0.001	0.001	0.001	0.001	0.001	0.001	0.001

C = coffee extract; CM = coffee extract and milk; 30 = 30 mg of extract; 40 = 40 mg of extract; 50 = 50 mg of extract; 60 = 60 mg of extract

S5. Impact of media incubation on gel morphology

In this study, the impact of media incubation on gel integrity was observed. The mini gels were formed from 500 μL of blank hydrogel solution in a 24-well plate. Hydrogels were prepared with based on D-factor shown in Table S2. In Figure S4, The top row's first three wells (left to right) contain 3X; then the subsequent three wells contain 2X formulations. Similarly, the bottom row's first three wells (left to right) contain 1X; then the next three wells contain 0.5X formulations (3.1 mg MBA, 6.25 mg APS). These mini-gels were soaked in 2 mL of DMEM media for 48 h.

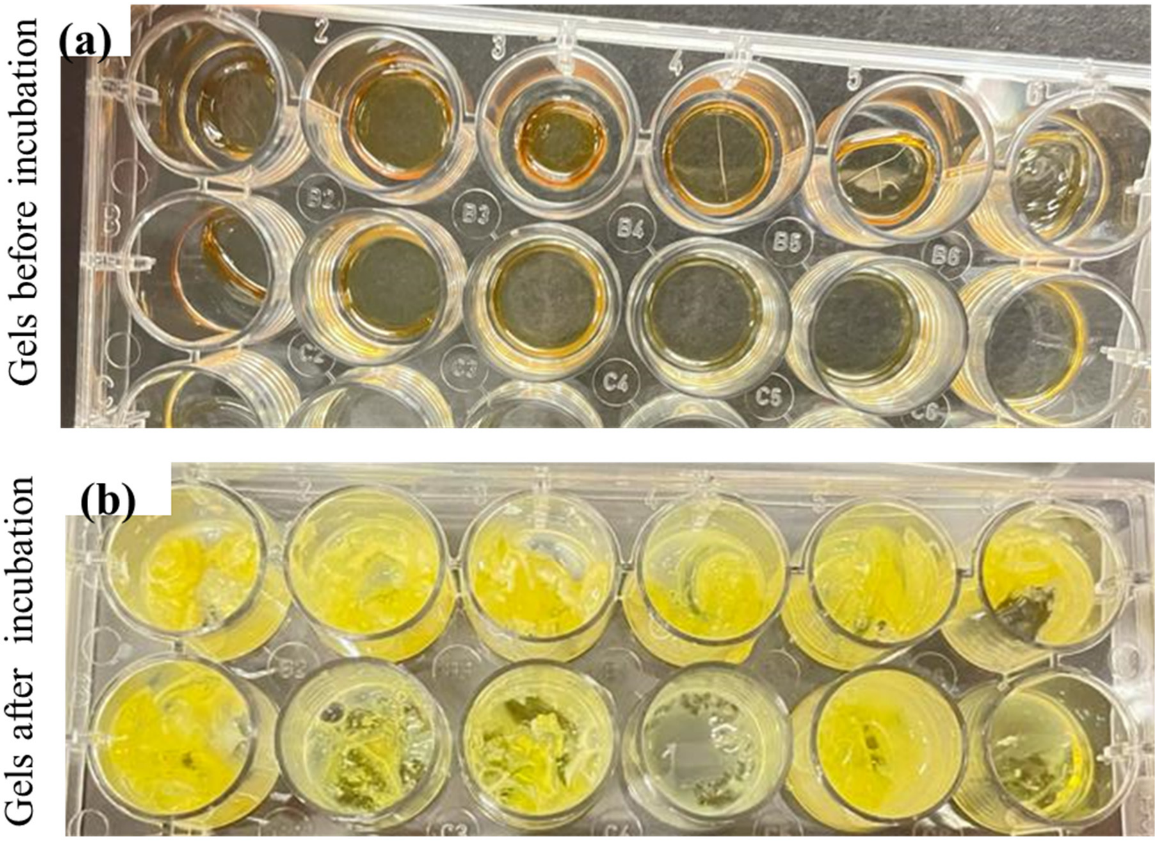

Figure S4. Effect of media (DMEM with 20% FBS) on hydrogel integrity. (a) Physical appearance of different gel batches formed in 24 well plates (b) Physical appearance of different hydrogels after soaking for 48 h in media.

References

1. 5 - Mathematical models of drug release. In *Strategies to Modify the Drug Release from Pharmaceutical Systems*, Bruschi, M.L., Ed.; Woodhead Publishing: 2015; pp. 63-86.
